# Competition in Biofilms between Cystic Fibrosis Isolates of *Pseudomonas aeruginosa* Is Shaped by R-Pyocins

**DOI:** 10.1128/mBio.01828-18

**Published:** 2019-01-29

**Authors:** Olubukola Oluyombo, Christopher N. Penfold, Stephen P. Diggle

**Affiliations:** a School of Life Sciences, University of Nottingham, Nottingham, United Kingdom; b School of Biological Sciences, Georgia Institute of Technology, Atlanta, Georgia, USA; University of Minnesota Medical School

**Keywords:** *Pseudomonas aeruginosa*, bacteriocins, biofilms, cystic fibrosis, pyocins

## Abstract

Pseudomonas aeruginosa is an opportunistic pathogen and the leading cause of morbidity and mortality in cystic fibrosis (CF) patients. P. aeruginosa infections are difficult to treat due to a number of antibiotic resistance mechanisms and the organism’s propensity to form multicellular biofilms. Epidemic strains of P. aeruginosa often dominate within the lungs of individual CF patients, but how they achieve this is poorly understood. One way that strains of P. aeruginosa can compete is by producing chromosomally encoded bacteriocins, called pyocins. Three major classes of pyocin have been identified in P. aeruginosa: soluble pyocins (S types) and tailocins (R and F types). In this study, we investigated the distribution of S- and R-type pyocins in 24 clinical strains isolated from individual CF patients and then focused on understanding their roles in interstrain competition. We found that (i) each strain produced only one R-pyocin type, but the number of S-pyocins varied between strains, (ii) R-pyocins were generally important for strain dominance during competition assays in planktonic cultures and biofilm communities in strains with both disparate R- and S-pyocin subtypes, and (iii) purified R-pyocins demonstrated significant antimicrobial activity against established biofilms. Our work provides support for a role played by R-pyocins in the competition between P. aeruginosa strains and helps explain why certain strains and lineages of P. aeruginosa dominate and displace others during CF infection. Furthermore, we demonstrate the potential of exploiting R-pyocins for therapeutic gains in an era when antibiotic resistance is a global concern.

## Abstract (precis)

A major clinical problem caused by Pseudomonas aeruginosa, is chronic biofilm infection of the lungs in individuals with cystic fibrosis (CF). Epidemic P. aeruginosa strains dominate and displace others during CF infection, but these intraspecies interactions remain poorly understood. Here we demonstrate that R-pyocins (bacteriocins) are important factors in driving competitive interactions in biofilms between P. aeruginosa strains isolated from different CF patients. In addition, we found that these phage-like pyocins are inhibitory against mature biofilms of susceptible strains. This highlights the potential of R-pyocins as antimicrobial and antibiofilm agents at a time when new antimicrobial therapies are desperately needed.

## INTRODUCTION

Pseudomonas aeruginosa is an opportunistic pathogen, capable of infecting different host species, including plants, insects, and mammals (1). It is intrinsically resistant to many classes of antibiotic and produces a range of tissue-damaging extracellular products such as exoenzymes and phenazine pigments, which aid dissemination and spread within a host (2). A major clinical problem caused by P. aeruginosa is chronic infection of the lungs in individuals with cystic fibrosis (CF), where it contributes significantly to morbidity over the lifetime of the patient and accelerates mortality (3). Strains that thrive in CF lung infections evolve in the lung environment over time, undergoing genomic mutations and rearrangements, which results in phenotypic variation within populations (4–6). The reasons why some epidemic and transmissible P. aeruginosa strains dominate and displace others during infection remain poorly understood, but one possibility is that intraspecies competition is driven by the production of bacteriocins known as pyocins.

Pyocins are ribosomally synthesized bacteriocins that are produced to kill competitors of the same species (7). The pyocins produced by P. aeruginosa are classified into three major types: S-, R-, and F-pyocins (8). The S-pyocins are large multidomain polypeptides that have a cognate immunity protein that binds to and inactivates the catalytic domain of the active pyocin (7). R- and F-type pyocins are defective prophages ancestrally related to P2 and lambda phages, respectively, which have differentiated into bacteriocins (8, 9). The R- and F-pyocins (collectively referred to as tailocins) possess no genetic material and therefore are nonreplicating (8, 10). Unlike the S-pyocins, the tailocins do not have a cognate immunity protein and resistance to them is mediated through incompatible lipopolysaccharide (LPS) (11). Antimicrobial activity of S- and R-type pyocins against planktonic cells of P. aeruginosa has previously been demonstrated (12–14), and pyocin S2 has been shown to be more effective against P. aeruginosa biofilms than aztreonam and tobramycin, both commonly prescribed antibiotics for chronic infections in CF patients (15). The killing mechanism of R-type pyocins is via a single-hit membrane depolarization, where one pyocin unit causes the death of a cell regardless of the number adsorbed to its surface (7, 8). The narrow specificity of killing has triggered interest in developing them as potent therapeutic alternatives to antibiotics (12).

While some work has been performed on the role of R-pyocins in biofilm formation (16), little work has focused on whether they influence strain competition and dominance in biofilms and ultimately *in vivo*. Here we investigate whether R-pyocins are important factors in competitive interactions between P. aeruginosa strains taken directly from CF lungs from different patients. We show that R1- and R2-type pyocins have potent activity against established biofilm communities of sensitive P. aeruginosa cells. By performing pairwise interactions between isolates, we identified two strains, A018 and A026, with reciprocated killing activity against each other. By making defined R-pyocin mutations in these strains, we determined that this activity was due to the production of different R-pyocin subtypes. We found that A026 produced an R1-type pyocin that was responsible for strain displacement and domination in both planktonic cultures and multicellular biofilms. More generally, our findings demonstrate a role for R-type pyocins in shaping ecological interactions and biofilm formation in P. aeruginosa. We also highlight the potential of R-pyocins as antimicrobial agents against P. aeruginosa at a time when new antimicrobial therapies are desperately needed.

## RESULTS

### Reciprocity of killing activity between paired *P. aeruginosa* CF isolates.

We first determined whether pyocins could play a role in ecological interactions between strains isolated from CF lungs by competing strains against each other. We isolated 24 P. aeruginosa CF strains from individual adult and pediatric patients, and we screened each one for pyocin killing using a spot assay to determine biological activity (17). We identified reciprocal activity between 9 strain pairs (A026/A014, A026/A018, A026/P003, A026/P013, A026/A007, A026/A024, A026/A032, A026/P010 and A014/A033), which was mediated by different subtypes of pyocin (see Table S1 in the supplemental material). We found 8 pairs with one common strain partner (A026). Reciprocated killing could have been due to any of the different pyocin subtypes; therefore, we investigated the class(es) of pyocins underpinning the strain antagonism that we observed.

10.1128/mBio.01828-18.1 TABLE S1 Interactions of 24 clinical P. aeruginosa strains using a spot test assay of biological activity. Vertical columns show the pyocin activities of each test strain, while the rows show each isolate as an indicator strain. Pairwise strain antagonisms are circled in red or blue and connected by red or blue lines. Red indicates pairwise activities having A026 as a competing member, while blue indicates reciprocal killing between A033 and A014. +, lethal activity shown by zone clearance; −, no activity. Download Table S1, PDF file, 0.1 MB. Copyright © 2019 Oluyombo et al. 2019 Oluyombo et al. This content is distributed under the terms of the Creative Commons Attribution 4.0 International license.

### Reciprocal killing of paired isolates is mediated by R-pyocins.

**FIG 1:**
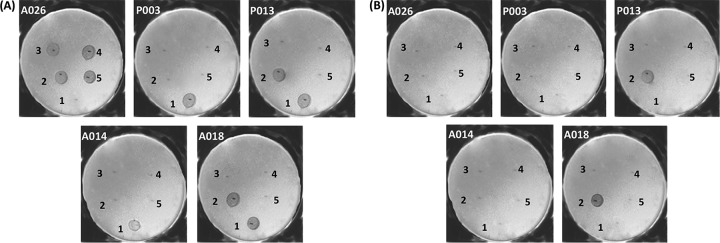
Spot test showing the biological activities of five representative clinical P. aeruginosa strains. Each plate is labeled with the indicator strain, while the numbers 1 to 5 represent test strains: 1 is A026, 2 is A014, 3 is P003, 4 is P013, and 5 is A018. In panel A, the cell extracts of the wild-type strains were used for the spot assay, while in panel B, the cell extracts of the null-R-pyocin mutant strains were used.

We screened for S- and R-pyocin subtypes in the 24 strains using primers specific to unique regions of each pyocin gene/operon (Table 1). We used 6 primer pairs for 6 subtypes of S-pyocins (S1, S2, S3, S4, S5, and AP41) and 3 for the R-pyocins (R1, R2 group, and R5). The R2 group of primers consisted of subtypes R2, R3, and R4 because they are essentially the same subtype, with 98% homology in their gene sequences. The profile of the distribution pattern is shown in Table 1. We found that the distribution of S-pyocin subtypes was varied, with most strains having one or more subtype. However, 2 strains (A026 and P015) did not have any of the screened S-pyocin genes. In contrast, the presence of an R-type pyocin gene operon was uniform, with each strain having a single R-pyocin subtype. We discovered that the R1 subtype was the most represented (15 isolates), whereas the least represented was R5 (1 isolate) (Table 1). As A026 did not possess any of the tested S-pyocin genes in its genome, but was involved in reciprocal killing of other strains (Table S1), we hypothesized that the killing using the spot test assay was due to one of the tailocins (R- or F-type pyocins). We amplified an R1-pyocin sequence from A026, which was in contrast to the R2-pyocin type found in all of the 8 pair members involved in the reciprocal interactions. These data were suggestive about the involvement of R-pyocins in reciprocal antagonism. To test this, we constructed null-R-pyocin deletion mutants (ΔR) in A026 and 4 strains (A014, A018, P003, and P013) that showed reciprocated antagonism against A026. We then repeated the spot test using the cell extracts of the ΔR strains and showed a loss of the biological inhibition in all 5 isolates against the protagonist strain (Fig. 1).

**TABLE 1:** Distribution of the six S-pyocin subtypes and three R-pyocin subtypes in the P. aeruginosa clinical isolates used in this study

Pyocin subtype	Distribution in clinical strain																							
	A007	A010	A014	A017	A018	A019	A024	A026	A031	A032	A033	A034	A035	A037	P003	P004	P006	P009	P010	P013	P015	P016	P018	P020
S-pyocins																								
S1			✓		✓										✓					✓				
S2	✓	✓	✓	✓	✓	✓	✓		✓		✓	✓	✓	✓	✓	✓	✓		✓	✓				✓
S3					✓				✓			✓	✓		✓	✓	✓		✓	✓				✓
S4	✓	✓	✓		✓	✓	✓		✓			✓	✓	✓	✓	✓	✓		✓	✓			✓	✓
S5	✓	✓	✓	✓	✓	✓	✓		✓	✓		✓	✓	✓	✓	✓	✓	✓	✓	✓			✓	✓
AP41	✓	✓	✓	✓			✓		✓		✓	✓	✓	✓	✓	✓	✓	✓	✓			✓		✓
R-pyocins																								
R1		✓				✓		✓	✓		✓	✓	✓	✓		✓	✓	✓			✓	✓	✓	✓
R2	✓		✓		✓		✓			✓					✓				✓	✓				
R5				✓																				

### R-pyocins mediate competition among strains existing in the same microenvironment.

**FIG 2:**
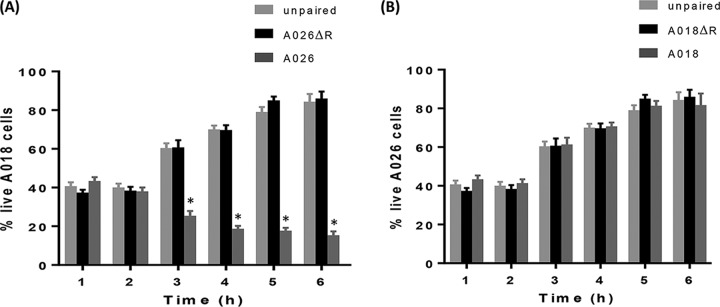
Planktonic cell competition assay in Transwell plates. (A) The A018 strain was grown alongside 3 different treatments (A026 wild type, A026 ΔR, or plain LB), and the percentage of surviving A018 cells in the population was determined over time. (B) The A026 strain was grown alongside 3 different treatments (A018 wild type, A018 ΔR, or plain LB), and the percentage of surviving A026 cells in the population was determined over time. Hourly estimation of the percentages was achieved by staining with BacLight LIVE/DEAD stain and viewing with a confocal laser scanning microscope. Live cell counts were performed in three fields for each reading. *, *P* < 0.001.

The role of R-pyocin in competition was previously reported in planktonic cultures of the laboratory strains PAK, PAO1, and PA14. PAK was outcompeted by the other two strains, which both produce R2-pyocin, unlike PAK, which contains a mutated *prf6* gene in its R-pyocin locus; thus, one competitor was weaker than the other (18). Here, we explored the involvement of R-pyocins in a new concept of head-to-head reciprocal killing which allows for a comparison of bidirectional pyocin activity and killing when either competing strain produces a lethal R-pyocin to the other. To complement the biological activities demonstrated in the spot assay (Fig. 1), we chose a representative competing pair, A026/A018, to study in further detail. We chose A026 (R1 producer) due to its prominence as a central competitor in 8 out of the 9 pairs, and we chose A018 (R2 producer) due to the comparative demographics of the two patients from whom A018 and A026 were isolated (27 and 32 years of age, respectively, with both diagnosed with CF as neonates). Both strains also had comparable growth rates, as determined by growth curves. We used Transwell membrane plates to compartmentalize the two strains, while allowing cell-free medium to pass through the membrane pores. We studied the wild-type (WT) and R-pyocin mutant (ΔR) versions of each strain in various head-to-head combinations. Figure 2 shows the survival rate of the WT version of each strain when cultivated in adjacent wells of either the WT or ΔR derivative of the competing strain. We used sterile growth medium as a control treatment. In paired interactions involving WT and ΔR strains of A026 and A018, we found that the dominant surviving strain was A026 WT (Fig. 2A and B), although toward the end of the experiment, approximately 20% of live A018 WT cells persisted in the population (Fig. 2A). As R-pyocins are induced by stress, we speculate that A018 was able to persist in the population because less A018 cells reduced competition with A026, potentially leading to reduced R-pyocin production and subsequent killing by A026.

### R-pyocins drive intraspecies competition in biofilms.

**FIG 3:**
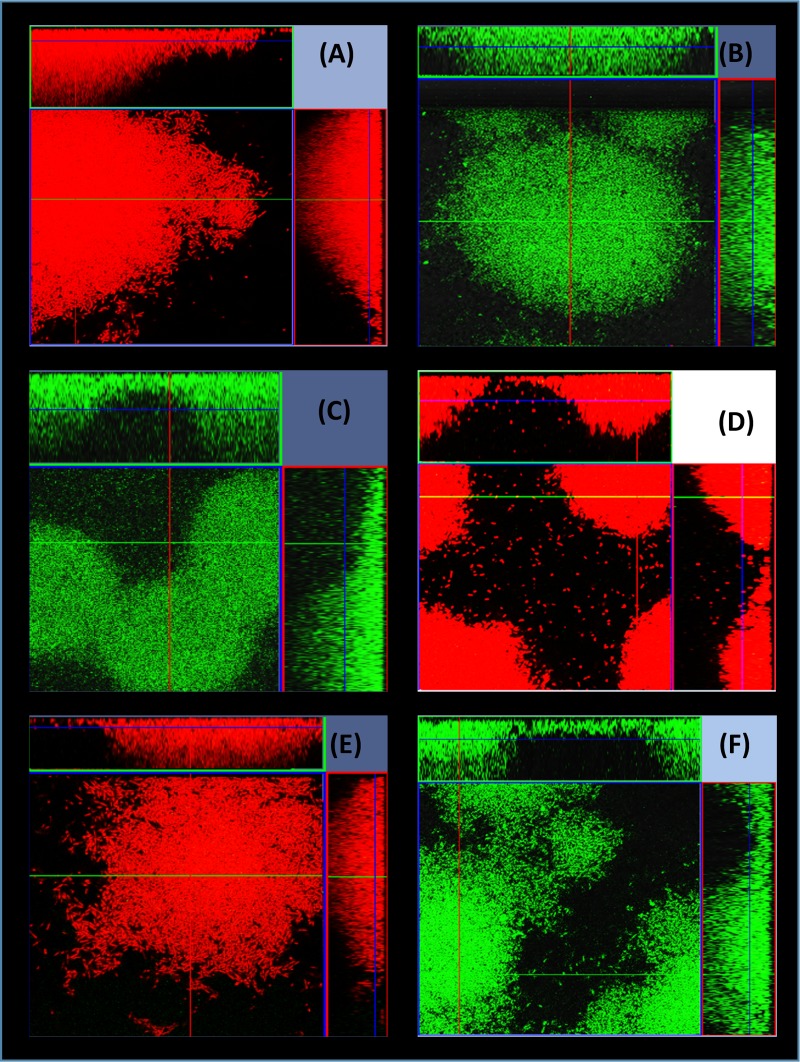
Strain competition in biofilms. Shown are 15-h biofilms developed in a microfluidic BioFlux device as a result of competition between A026 and A018. A026 dominated in the biofilm competition of A018 versus A026 and A018 ΔR versus A026 regardless of the fluorophore (mCherry or GFP) used to tag either strain (A to D). However, A018 dominated when in competition with the null-R mutant of A026 (A026 ΔR) (E and F). (A) A026_mCherry versus A018_GFP. (B) A026_GFP versus A018_mCherry. (C) A026_GFP versus A018 ΔR_mCherry. (D) A026_mCherry versus A018 ΔR_GFP. (E) A026 ΔR_GFP versus A018_mCherry. (F) A026 ΔR_mCherry versus A018_GFP.

**FIG 4:**
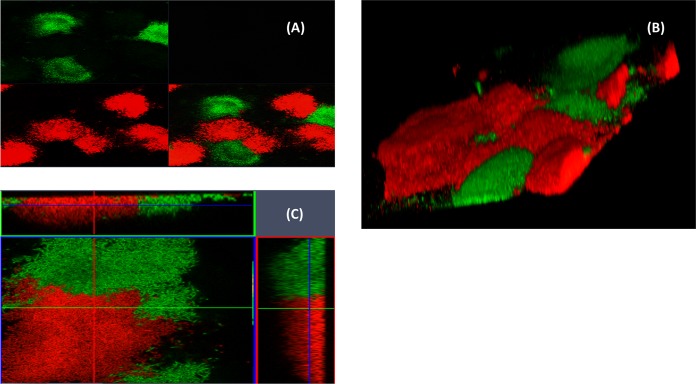
Fifteen-hour biofilms showing the distribution of two strains. (A and B) Biofilms of mixed cultures of A018 ΔR_GFP and A026 ΔR_mCherry. Panel A is a split image of the fluorescent green and red channels at ×10 magnification showing a wider distribution of the strains as they form microniches, while panel B shows a closer view of the same biofilms at ×63 magnification. Panel C shows a biofilm of wild-type strains A018_GFP and P013_mCherry, which both have the same subtypes of S- and R-pyocins.

R-pyocins were shown to be important for the competition of planktonic cells in strains occupying adjacent compartmentalized environments (Fig. 2), but this does not represent the conditions found in ecosystems in which strains compete side by side in biofilms for survival. In order to investigate the effect of R-pyocins on two interacting strains that coexist in a common ecological niche, we competed strains in biofilms. We achieved this by mixing A018 and A026 differentially labeled with green fluorescent protein (GFP) or mCherry in microfluidic channels of a BioFlux device and allowed them to form biofilms over time. In total, we used six combinations of labeled strains (Fig. 3). When A026 WT labeled with either GFP (A026_GFP) or mCherry (A026_mCherry) was incubated with A018 WT labeled with the contrasting fluorophore, we found that A026 always outcompeted A018 (Fig. 3). However, when A018 WT was mixed with A026 ΔR, A018 outcompeted A026 ΔR (Fig. 3). When R mutants of both strains (A018 ΔR and A026 ΔR) were competed against each other, both strains were able to coexist within biofilms, but they were clearly spatially separated, enabling each to form individual pockets within biofilms that were strain specific (Fig. 4A and B). Since inactivating R-pyocins in strains with different R-pyocin types abolished competition, we explored whether 2 strains with matching R-pyocin types could coexist. We mixed 2 strains, A018 and P013, which were tolerant of each other in the spot test assay (Table S1) and possessed the same R- and S-type pyocins (Table 1), to study the patterns of biofilm formation. We found that both strains could coexist and produced biofilms with a similar architecture to the R-mutant biofilms of A018 and A026 (Fig. 4C).

### R-pyocins demonstrate antibiofilm properties in established biofilms.

**FIG 5:**
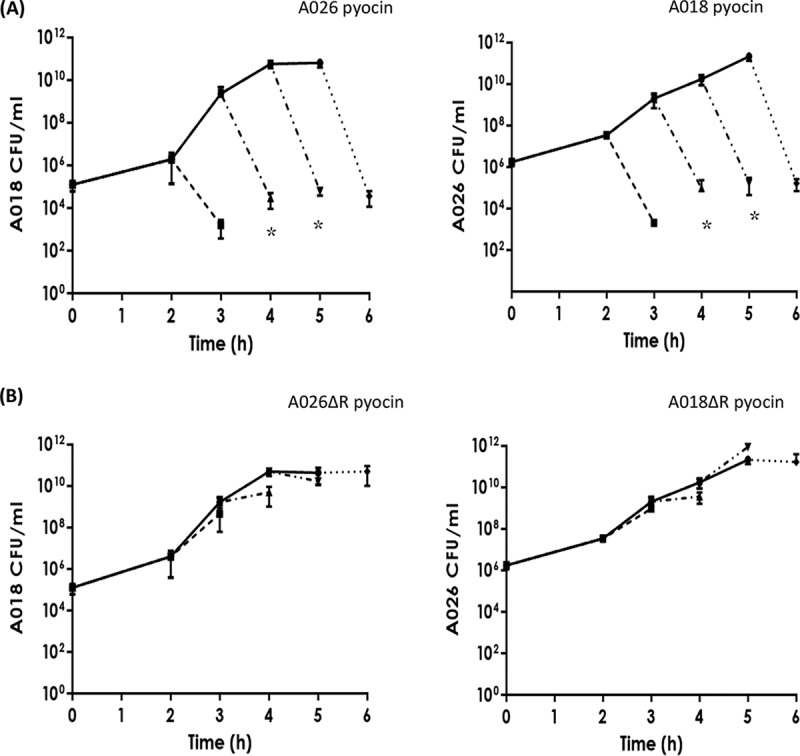
Single static treatment of biofilms of A018 or A026 grown on polystyrene beads using R-pyocins of a competitor (A026 or A018, respectively). Hour 0 readings are the CFU/ml values of the 24-h biofilm grown on the beads. In one set of beads, growth was allowed to proceed unhindered (unbroken lines), while in the other set, three beads were harvested every hour, their biofilms were treated for 1 h using purified R-pyocins, and the CFU count was recorded after treatment (broken lines). Beads in panel A were treated with R-pyocins from the wild type of competitor, while the ones in panel B were treated with cell extracts from R-pyocin mutants. *, *P* < 0.05.

**FIG 6:**
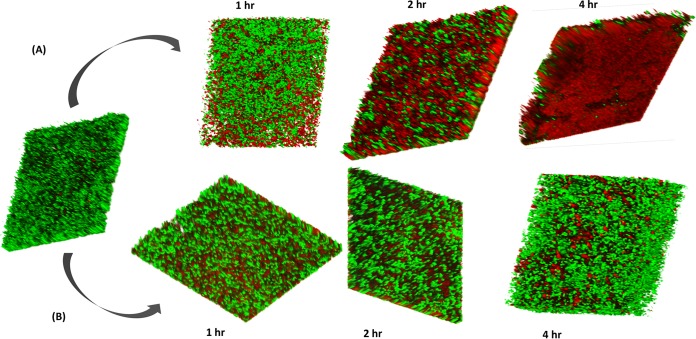
Antibiofilm efficacy of R-pyocins. A 15-h biofilm of A018 was treated with R-pyocins extracted from A026 (A) and A026 ΔR (B). A significant portion of the biomass was killed after 2 h and full-depth lethal effects on the biomass was achieved after 4 h. This effect was absent in the control experiment, which utilized the R-pyocins of A026 ΔR.

The loss of biological activity in the R-pyocin mutant spot test suggested the involvement of this class of pyocin in biofilm killing activity. We induced R-pyocin production in either A018 or A026 to purify high pyocin yields and tested these against biofilms. We subjected biofilms of A018 or A026 grown on polypropylene beads to a single treatment of R-pyocins from the competitor strain for one hour, and we counted viable cells before and after treatment over time. We found that R-pyocins of each strain caused a significant reduction of viable cell counts of the competing strain (Fig. 5A). However, extracts from the R-pyocin mutants of either strain did not produce any significant differences in the viable cell count of the competitor compared to the untreated population (Fig. 5B). We also used a BioFlux system to achieve a longer exposure time to R-pyocins and a dynamic flow. We found that treatment of mature biofilms with R-pyocins resulted in significant diminishment of live cell populations over time, with a corresponding increase in the number of dead cells. There was a progressive loss of the number of viable cells in biofilms, and full-thickness biomass eradication was achieved within 4 h of starting R-pyocin treatment using purified R-pyocins of wild-type strains. (Fig. 6A shows antibiofilm activity of A026 R-pyocin against A018 biofilm.) In contrast, the extract purified from the R-pyocin mutants showed no adverse effect on the biofilm growth and maturation of the competing strain (Fig. 6B shows A026 ΔR extract against A018 biofilm). The results were similar when A026 biofilms were treated with the R-pyocins/extracts from A018 and A018 ΔR, respectively.

## DISCUSSION

A key clinical problem and research priority in CF is how to treat and eradicate P. aeruginosa from the CF lung, as well as how to prevent pulmonary exacerbations (3, 19). This has proven incredibly challenging and is likely due to the overwhelming phenotypic and genomic diversity that we now know evolves within *in vivo* P. aeruginosa populations (4–6). Some epidemic strains, such as those from the Liverpool (LES) and Denmark (DK2) epidemics, have become extremely successful, infecting many different patients across multiple geographic locations. How these transmissible and persistent P. aeruginosa lineages displace and prevent the colonization of other strains remains poorly understood, but elimination of competing strains is one potential survival strategy. Competing against genetically similar strains is an important means of self-preservation, because available nutrients and space in an ecological niche are more likely to be competed for between highly related strains.

Certain factors have previously been suggested to influence strain succession and persistence. Production of a compound from environmental pseudomonads possessing a homologue of the thioquinolobactin gene cluster was attributed to strong antagonistic effects on clinical isolates of P. aeruginosa (20), while the role of temperate phages in improving competitive fitness in CF lungs was recently reported (21). In addition, over 90% of known strains of P. aeruginosa are able to produce pyocins (7, 8). These bacteriocins often possess a narrow spectrum of antimicrobial activity against closely related strains and so are likely to be involved in interstrain competition. Recently it has been shown that strains of P. aeruginosa isolated from CF lungs are particularly susceptible to R-pyocins, mainly due to their LPS architecture with nontypeable or inaccessible O serotypes (22).

In this study, we provide support that R-type pyocins help shape ecological interactions between CF isolates of P. aeruginosa in biofilms and that R-pyocins have potential as antimicrobial agents. Specifically, we found that (i) individual P. aeruginosa strains taken from different CF patients produced only one R-pyocin type, whereas the number of S-pyocin types produced by any one strain varied, (ii) R-pyocins were crucial for strain dominance during competition assays in planktonic cultures and within biofilms, as well as where variations in both R and S subtypes occurred in the competing strains, and (iii) purified R-pyocins from different strains demonstrated significant antimicrobial activity against established biofilms.

We began our study by isolating 24 P. aeruginosa strains from 24 different CF patients, and we identified several isolates with reciprocated antagonism in pairwise interactions. Deletion mutagenesis of essential regions of R-pyocin genes of each strain revealed that the antagonistic activity was due to the production of R-pyocins. While the number of S-subtype pyocins differed per strain, ranging from zero to all six tested S-type pyocins (Table 1), each strain had only one R-subtype. With little genetic redundancy in the bacterial genome, the incorporation of an extra S-pyocin gene will be more amenable to genetic restructuring than the assembly of multiple R-type operons. Indeed, many P. aeruginosa strains possess extra “orphan” immunity genes to the S-type pyocins, presumably creating increased pyocin resistance with little extra cost (23). The diversity of S-type pyocins displayed by some of the clinical isolates did not proportionally translate to better survival in the face of competition. Contrariwise, the strain A026, which demonstrated the highest competitive ability, did not have any of the six screened S-pyocin types (Table 1).

The abolishment of killing activity in some of the clinical isolates was only achieved in R-pyocin mutants, even if a strain had an excess of S-pyocin genes, highlighting a role of R-pyocins in strain survival (Fig. 1). However, it is important to note that killing patterns between isolates using the spot assay (Fig. 1 and Table 1; Table S1) did not always show a clear and obvious role for R-pyocin killing. For example, A031 (an R1 producer) was highly sensitive to pyocin extracts from other strains but also killed other R1 producers (Table S1). This suggests that A031 kills in an R-pyocin-independent manner, and indeed, F-pyocins and/or phage are also likely to be important in P. aeruginosa strain competition. Strains can also become sensitive to their own R-pyocins through altered LPS (23), and this further complicates the picture. More work is required to unravel all the factors that drive interactions between P. aeruginosa strains.

R-pyocins have previously been shown to confer a growth advantage to producing strains *in vitro* during competition of planktonic cells (11, 18, 24) and promote antimicrobial activity against P. aeruginosa *in vivo* using murine models (12). However, any proposed role in strain competition *in vivo*, or the applicability of R-pyocins as potential antimicrobials, hinges on their ability to combat biofilms, the preferred mode of P. aeruginosa growth in chronic infections such as in the CF lung and chronic wounds. We demonstrated a direct association of R-pyocins with antibiofilm efficacy. Although a previous study showed that subinhibitory concentrations of cell-free culture supernatants produced an enhancement of biofilm formation of susceptible strains (16), we found that exposure of two competing strains to each other ensured R-pyocin-dependent killing in biofilms over time.

Our study also highlights the potential of using R-pyocins as an antibiofilm strategy and that R-pyocins can effectively eradicate biofilms composed of a susceptible strain (Fig. 6). Exposure of live cells, planktonic or biofilm, to extracted R-pyocins (Fig. 1 and 6, respectively) results in killing in the absence of a live competing strain (Fig. 2A). Therefore, R-pyocins could be a useful alternative for antibiotics or phage therapy for infections involving biofilms or for biofilms growing on medical devices such as catheters. In contrast to phages, the absence of a capsid containing genetic material in the structure of the R-pyocins nullifies their ability for self-replication, and they also have highly specific killing action (8).

In summary, R-pyocins are a class of tailocins that are likely to be important in shaping P. aeruginosa populations during chronic biofilm-driven infections, such as those found in the CF lung and in chronic wounds. Mutation of the R-pyocin locus was a major factor that eliminated competition between strains in our study, and coexistence of strains was achieved when biofilms of R-pyocin mutants of competing strains were cultivated together. They therefore could have particular relevance in niches where competition is central to survival. More work is required in the future as to the mechanisms that regulate R-pyocin production and their role in shaping P. aeruginosa ecology and their potential role as therapeutic agents.

## MATERIALS AND METHODS

### Bacterial strains, plasmids, media, and culture conditions.

We used 24 P. aeruginosa isolates taken from CF sputum samples (14 adult and 10 pediatric patients) and Escherichia coli (S17-1 [25] and DH5α [26]) strains in this study (Table 2). We routinely cultured our strains in lysogeny broth (LB) agar at 37°C and cultivated the biofilms of our P. aeruginosa clinical strains in minimal medium M9 broth supplemented with 0.2% citrate. (M9 broth is made up of basal salt [68 g/liter Na_2_HPO_4_, 30 g/liter KH_2_PO_4_, and 5 g/liter NaCl], 10^−2^ M NH_4_Cl, 10^−4^ M CaCl_2_, and 10^−3^ M MgSO_4_⋅7H_2_O.) Using tetracycline (Tc) at 25 µg/ml, we maintained and/or selected our plasmids in E. coli S17-1, while our P. aeruginosa transconjugants were selected on plates containing 150 µg/ml Tc plus 15 μg/ml nalidixic acid.

**TABLE 2:** Strains, plasmids, and primers used in this study

Strain, plasmid, or primer	Properties	Reference or source
Strains		
*E. coli*		
S17-1 λ*pir*	*galU galK rpsL*(Str^r^) *endA1 nupG thi pro hsdR* *hsdM^+^ recA* (RP4-2 Tc::Mu Km::Tn*7*) λpir	25
DH5α	F^−^ ϕ80*lacZ*ΔM15 Δ(*lacZYA-argF*) *U169 recA1* *endA1 hsdR17*(r_K_^–^ m_K_+) *phoA supE44* λ^–^ *thi-1* *gyrA96 relA1*	26
*P. aeruginosa*		
Clinical isolates^*a*^	A007, A010, A014, A017, A018, A019, A024, A026, A031, A032, A033, A034, A035, A037, P003, P004, P006, P009, P010, P013, P015, P016, P018, P020	Nottingham collection
Null-R-pyocin mutants of clinical isolates	A014 ΔR, A018 ΔR, A026 ΔR, P003 ΔR, P013 ΔR	This study
mCherry-tagged isolates containing pME6032-ptac::mCherry	A026_mCherry, A018_mCherry, A026 ΔR_mCherry, A018 ΔR_mCherry, P013_mCherry	This study
GFP-tagged isolates containing pME6032-ptac::GFP	A026_GFP, A018_GFP, A026 ΔR_GFP, A018 ΔR_GFP, P013_GFP	This study

Plasmids		
pME3087	Suicide vector; ColE1 replicon, IncP-1, Mob; Tc^r^	28
pOO1	pME3087 bearing construct gene with deletion of PA020 and PA021 (pME3087::ΔPA0620-21); Tc^r^	This study
pME6032-ptac::EGFP	Shuttle vector between *E. coli* and *P. aeruginosa* containing *lacI*^q^*-Ptac*::EGFP; Tc^r^	Nottingham collection
pME6032-ptac::mCherry	Shuttle vector between *E. coli* and *P. aeruginosa* containing *lacI*^q^*-Ptac*::mCherry; Tc^r^	Nottingham collection

Primers (bp)^*b*^		
Gene amplification		
S1-F (518)	TTCAACTCTACAACTGTCACG	This study
S1-R	TTCCATTTCCCTGTCGAGG	
S2-F (770)	TTCGATGGTTATTACACATGTGC	This study
S2-R	AAGGCATTGTTTGCAGTCTGC	
S3-F (320)	TGAATGGAGAAGAAGCTGATCG	This study
S3-R	TCTCTCGTCTCAAATGGTTTCC	
S4-F (236)	AGAAGGCAATGGGAAGATGTG	This study
S4-R	AAGCATCTTCCTCTGTACTCTC	
S5-F (415)	ATACGAGGTTCCCCCTATCG	This study
S5-R	AACAAGCTGCTGAAAAGGGTAC	
AP41-F (870)	AATTGTCGATGGCGAACTGG	This study
AP41-R	ATTGAAACACTGCCGACATCG	
R1-F (441)	ATGATTTTTTTCCATGCCGCCACG	This study
R1-R	TCAGGGGGTGATGAGCGATTGG	
R2-F (257)	ATGCCGATGCTTCGATTAC	This study
R2-R	AAACCTCTCGCAAGGAGG	
R5-F (140)	TGGAATCGTCAACCGCTCGCTG	This study
R5-R	TGGTGCTGACGCTGACATCTGC	
Null-R-pyocin mutant generation		
L-0619H3 (454)	TCAAGCTTAGCCGACTGCTGCCGCCAAAC	This study
R-0619	AGCCGATGCCGAACGGTCAGGTGTCTGCTC	
L-0622 (637)	CGTTCGGCATCGGCTCGTCTATCTACCTGG	This study
R-0622BHI	ATGGATCCCCACAGGCGATAGCCATCGTCG	

a Listed are the 14 adult strains (A007 through A037) and 10 pediatric strains (P003 through P020).

b The size in base pairs for each primer pair is given in parentheses.

### PCR conditions.

All primers used in this study were designed by using the P. aeruginosa Genome Database as a reference (http://www.pseudomonas.com) (27) and are listed in Table 2. Our PCR volume of 50 µl comprised 2.5 µl each of the primers (10 mM) and the template, 10 µl each of buffer and GC enhancer, 1 µl of deoxynucleoside triphosphates (dNTPs [10 mM]), 0.5 µl of Q5 high-fidelity polymerase, and 21 µl of deionized water. The PCR conditions we used were 98°C for 30 s, followed by 30 cycles of 98°C for 10 s, 58°C for 30 s, and 72°C for 30 s/kb, followed by a final extension at 72°C for 2 min.

### Spot assay for pyocin activity.

Using 2 µl of the overnight cultures of our P. aeruginosa isolates (adjusted to an optical density at 600 nm [OD_600_] of 0.5 ± 0.02), we inoculated 5 ml of cooled soft top (0.4%) agar. We poured this mixture onto LB agar plates to produce an overlay of the indicator strain. Next, we vortexed equal volumes of the overnight broths of the test strains and chloroform. We centrifuged the vortexed mixture to obtain cell extracts from the supernatant. We spotted 7-µl drops from the supernatant of individual test strains onto the agar overlay indicator strains, allowing the spots to dry and incubating at 37°C overnight. Clear zones of growth inhibition indicate pyocin-dependent lysis of the susceptible strains.

### R-pyocin mutant generation.

We generated R-pyocin mutants by deleting the genes coding for the R-pyocin tail fiber and its chaperone assembly gene (PA0620 and PA0621 in the PAO1 reference strain). We achieved this using a double homologous recombination method with the help of our deletion gene construct. Our 1,076-bp gene construct was generated from a two-stage PCR. In the first-stage PCR, we amplified the upstream and downstream genes PA0619 and PA0622 (flanking our genes of interest, PA0620 and PA0621) separately using primer pairs L0619H3/R0619 and L0622/R0622BHI. This first stage generated 454- and 637-bp amplicons, respectively. In the second stage, we used the two amplicons from the first stage as the templates, bringing the engineered 15-bp complementary ends together by splicing of overlapping extensions using the end primers L0619H3 and R0622BHI. Next, we cloned the gene construct into HindIII/BamHI-digested pME3087 (28) to produce plasmid pOO1, which was used to transform E. coli S17-1 strains. We then integrated pOO1 into P. aeruginosa chromosomes by conjugating P. aeruginosa strains with transformed S17-1 cells. We selected our P. aeruginosa transconjugants using agar plates containing 150 µg/ml Tc plus 15 μg/ml nalidixic acid. We picked individual colonies from our selective plates and cultured them in overnight broths without antibiotics in order to enrich for tetracycline-sensitive cells. We then made fresh cultures (1:100 dilution) from the overnight broth and incubated until they reached an OD_600_ of 0.1 ± 0.05. We added tetracycline and carbenicillin in turn at 1-h intervals to achieve final concentrations of 20 µg/ml and 2,000 µg/ml, respectively. Then we washed our cells using sterile LB and repeated the enrichment process (in plain and antibiotic broths). After washing the cells, we plated out their dilutions (10^−5^ to 10^−8^) on LB agar to harvest individual colonies. We performed a replica plating of individual colonies using LB and 25-µg/ml Tc agar plates. This step selected for unmarked mutants with double crossovers, and since they were sensitive to tetracycline, we picked them from the LB plates. We further confirmed the deletion mutation by PCR using primer pair L0619H3 and R0622BHI, thus, recovering our 1,076-bp deletion gene construct. We tested for the successful attenuation of pyocin activity by performing spot assays using cell extracts from these null-R-pyocin mutants and comparing the results with cell extracts from wild-type bacteria.

### Induction and purification of R-pyocins from *P. aeruginosa*.

The method used for induction and purification is a modified method adopted from reference 29. Briefly, starting with 1:100 starter culture from an overnight P. aeruginosa broth, we achieved log-phase growth (OD_600_ of ∼0.25) in 2 liters of G-medium (20 g/liter sodium glutamate, 5 g/liter glucose, 2.23 g/liter Na_2_HPO_4_, 500 mg/liter yeast extract, 250 mg/liter KH_2_PO_4_, and 100 mg/liter MgSO_4_⋅7H_2_O). We then added mitomycin C to a final concentration of 3 µg/ml. After incubating the culture for a further 3 h, we removed the cell debris by centrifugation at 17,000 × *g* for 1 h at 4°C. To the supernatant, we added 4 M (NH_4_)_2_SO_4_, titrating this at a rate of 1 ml/min with continuous stirring at 4°C. After overnight storage at 4°C, we centrifuged the suspension at 22,000 × *g* for 1 h to harvest the pellets containing the R-pyocins. We resuspended the pellets in TN50 buffer (50 mM NaCl and 10 mM Tris HCl adjusted to pH 7.5) and further performed ultracentrifugation at 65,000 × *g* and 4°C for 1 h to further concentrate the R-pyocins. We resuspended and stored our R-pyocin-rich pellets in 2 ml TN50 buffer at 4°C.

### Transwell membrane plate method for planktonic cell activity.

We used a standard 6-well plate with a Transwell polyester membrane (Corning) to study cell-free interactions between P. aeruginosa isolates placed on either side of the membrane. The Transwell units are made up of coupled multiwell plates. Each couple has an outer well set up in a plate of six wells and individual insert wells. The base of the insert has a permeable support through which cell-free exchange of culture medium takes place. The membrane pores are 0.4 µm in diameter, through which soluble and particulate pyocins (R-pyocin dimension of 120 nm by 5 nm [30]) are freely exchanged. Cultures of the two strains are grown separately, adjusted to comparable optical densities, and placed in separate wells of each Transwell couple. We started the initial cultures of either isolate at an OD_600_ value of 0.05 ± 0.01, with each pair set up in triplicates using a 2.5-ml volume in each well. We incubated the culture plates with gentle shaking (60 rpm) at 37°C for 2 h. Thereafter, we took 100-µl samples from either well and washed them twice with phosphate-buffered saline (PBS). We then stained the cells using BacLight LIVE/DEAD stain by incubating them in the stain for 30 min at 37°C. We used this staining method for each cell population on either side of the Transwell membrane and viewed them under the confocal microscope. We carried out two sets of confocal studies. In the first set, we viewed the cells in their native state with minimal disturbance by skipping the PBS washing step. The aim of the second set was cell counting, so we washed and diluted out the cultured cell populations. We counted three fields for each cell culture at ×60 magnification. We included two controls: these were null-R-pyocin mutants of either strain paired up with the wild type of the competitor or unpaired (i.e., the free-growing wild type of either strain with plain LB medium in the adjacent well). Using the live and the total cell counts, we calculated the percentage of live cells in different fields over time.

### Biofilm development on 3D pearl beads and pyocin treatment.

We used minimal medium M9 broth for our bead biofilm cultivation. These beads, in hollow cylinder shapes, were made of polystyrene and had an approximate total surface area of 2.6 cm^2^. Starting with a 1:100 dilution of culture, we suspended these sterile beads in 100 ml of fresh M9 culture in a 500-ml flask. The incubation conditions we employed were 37°C with shaking at 80 rpm for 24 h. After 24 h, we harvested the beads and resuspended them in fresh broth that had been grown to the log phase. At this stage, we started the timing of the experiment, taking the start of the new cultures as 0 h. At 2 h, we commenced hourly harvesting of six beads from the lot. The harvested beads were gently rinsed twice in PBS to rid them of planktonic cells. We divided these six beads into two sets of three. We used the first set for CFU cell counting by vortexing them in 1 ml PBS and plating them in dilutions. We used the second set to study R-pyocin treatment by suspending the beads in purified R-pyocins for 1 h and thereafter performing the cell count. Our controls included a third set of beads that were treated with cell extracts from null-R mutant derivatives of each isolate.

### Growing monoculture and mixed-culture biofilms in a microfluidic system.

We employed the microfluidic system of BioFlux 200 (Fluxion Biosciences, Inc., Alameda, CA) to cultivate our biofilms. The system comprised twin wells (inflow and outflow) connected by microchannels for cultivating biofilms. As an initial step, we primed the channels with 100 µl of LB medium from the inflow well with a share pressure setting of 2 dynes/cm^2^ for 3 min. Thereafter, we seeded the microchannels from the outflow well using fresh cultures at the mid-log growth phase (OD_600_ of approximately 0.05) by applying a back pressure of 0.5 dyne/cm^2^ for 2 s. We then incubated the setup on the heating plate at 37°C for 30 min without flow to enhance adhesion of the cells to the channels. We coupled the microfluidic system for overnight incubation (approximately 15 h) with pressure and temperature settings of 0.25 dyne/cm^2^ and 37°C, respectively. The strains that we used to seed the microchannels and cultivate our biofilms were previously tagged with either green fluorescent protein (GFP) or mCherry. Our biofilms were cultivated as either monocultures or mixed cultures. Our monocultures consisted of A026_GFP, A026_mCherry, A018_GFP, A018_mCherry, A026 ΔR_GFP, A026 ΔR_mCherry, A018 ΔR_GFP, A018 ΔR_mCherry, P013_GFP, P013_mCherry, P013 ΔR_GFP, and P013 ΔR_mCherry, while the mixed cultures consisted of differentially labeled pairs: i.e., A026_mCherry/A018_GFP, A026_GFP/A018_mCherry, A026_GFP/A018 ΔR_mCherry, A026 ΔR_mCherry/A018 ΔR_GFP, and A018_mCherry/P013_GFP, among others. After 15 h, we viewed our biofilms using a confocal laser scanning microscope (Zeiss LSM 700).

### R-pyocin treatment of biofilms in a microfluidic system.

We studied a dynamic flow of pyocin treatment on biofilms cultured in the BioFlux 200 microfluidic system. To achieve this, we cultivated 15-h biofilms, staining the cells as they multiplied, by adding the BacLight LIVE/DEAD stain to the 10% LB broth used to cultivate the biofilm. We then treated the 15-h mature biofilms with purified R-pyocins by changing the medium to the TN50 buffered R-pyocin suspension mixed with the LIVE/DEAD stain. We maintained the same treatment conditions (temperature and pressure) for a further 5 h. We included two controls in the study: these were biofilms treated with null-R mutant extracts and untreated biofilms.
