# Supplementary material for: Competition in Biofilms between Cystic Fibrosis Isolates of Pseudomonas aeruginosa Is Shaped by R-Pyocins
Source: mBio. 2019 Jan 29;10(1):e01828-18. doi: 10.1128/mBio.01828-18 (PMC6355985; doi:10.1128/mBio.01828-18)
Supplement: TABLE S1 [file mBio.01828-18-st001.pdf]

INDICATOR STRAINS	PYOCINS OF TEST STRAINS																							
	A007	A010	A014	A017	A018	A019	A024	A026	A031	A032	A033	A034	A035	A037	P003	P004	P006	P009	P010	P013	P015	P016	P018	P020
**A007**	-	-	-	-	-	-	-	+	-	-	-	-	-	-	-	-	-	-	-	-	-	-	-	-
**A010**	+	-	+	-	+	-	+	-	+	+	+	-	-	-	-	+	+	-	-	+	+	-	-	+
**A014**	+	-	-	-	+	-	-	+	-	+	+	-	-	-	-	-	-	-	-	+	-	-	-	-
**A017**	+	-	+	-	+	-	+	-	+	+	+	-	+	-	-	+	-	-	-	+	+	-	-	+
**A018**	-	-	-	-	-	-	-	+	-	-	-	-	-	-	-	-	-	-	-	-	-	-	-	-
**A019**	+	-	+	-	+	-	+	-	+	+	+	-	+	-	-	+	-	-	-	+	+	-	-	+
**A024**	+	-	-	-	+	-	-	+	-	+	-	-	-	-	-	-	-	-	-	+	-	-	-	-
**A026**	+	-	+	-	+	-	+	-	+	+	+	-	-	-	+	+	-	-	+	+	+	-	-	-
**A031**	-	-	-	-	-	-	-	-	-	-	-	-	-	-	-	-	-	-	-	-	-	-	-	-
**A032**	-	-	-	-	-	-	-	+	-	-	-	-	-	-	-	-	-	-	-	-	-	-	-	-
**A033**	+	-	+	-	+	-	+	-	-	+	-	-	-	-	-	-	-	-	-	-	-	-	-	+
**A034**	-	-	-	-	-	-	-	-	-	-	-	-	-	-	-	-	-	-	-	-	-	-	-	-
**A035**	+	-	+	-	+	-	+	-	+	+	+	-	-	-	-	-	-	-	-	-	+	-	-	+
**A037**	+	-	+	-	+	-	+	-	+	+	-	-	-	-	-	-	-	-	-	-	-	-	-	+
**P003**	-	-	-	-	-	-	-	+	-	-	-	-	-	-	-	-	-	-	-	-	-	-	-	-
**P004**	-	-	-	-	-	-	-	-	-	-	-	-	-	-	-	-	-	-	-	-	-	-	-	-
**P006**	+	-	+	-	+	-	+	-	+	+	-	-	-	-	-	-	-	-	-	-	-	-	-	+
**P009**	+	-	+	-	+	-	+	-	-	+	-	-	-	-	-	-	-	-	-	-	+	-	-	+
**P010**	-	-	-	-	-	-	-	+	-	-	-	-	-	-	-	-	-	-	-	-	-	-	-	-
**P013**	-	-	-	-	-	-	-	+	-	-	-	-	-	-	-	-	-	-	-	-	-	-	-	-
**P015**	+	-	+	-	+	-	+	-	+	+	+	-	-	-	-	-	-	-	-	-	-	-	-	+
**P016**	+	-	+	-	+	-	+	+	+	+	+	-	-	-	-	-	-	-	-	-	-	-	-	+
**P018**	+	-	+	-	+	-	+	+	+	+	+	-	-	-	-	-	-	-	-	-	-	-	-	-
**P020**	+	-	-	-	+	-	-	+	-	+	-	-	-	-	-	-	-	-	-	+	-	-	-	-

Table S1. Interactions of 24 clinical *P. aeruginosa* strains using a spot test assay of biological activity. Vertical columns show the pyocin activities of each test strain while the rows show each isolate as an indicator strain. Pairwise strain antagonism are represented as dumb bells. Red dumb bells for pairwise activities having A026 as a competing member while the blue dumb bell represents reciprocal killing between A033 and A014 (+ = lethal activity shown by zone clearance, **-** = no activity).
